# Re-emergence of Conventional Drug Susceptibility Amid Persistent Fluoroquinolone Resistance in Enteric Fever

**DOI:** 10.7759/cureus.113559

**Published:** 2026-07-28

**Authors:** Mallika Sengupta, Sreeram R S, Sunitaj Parvin, Shiv S Chatterjee, Ujjala Ghoshal

**Affiliations:** 1 Microbiology, All India Institute of Medical Sciences Kalyani, Kalyani, IND

**Keywords:** antimicrobial susceptibility, enteric fever, fluoroquinolone resistance, salmonella paratyphi, salmonella typhi

## Abstract

Background

Fluoroquinolone resistance among *Salmonella* isolates causing enteric fever has emerged as a major therapeutic challenge in India. The present study evaluated the clinical profile, antimicrobial susceptibility patterns and genomic mechanisms of fluoroquinolone resistance among *Salmonella* isolates from a tertiary care centre in Eastern India.

Methods

This retrospective observational study included 30 patients with blood culture-confirmed enteric fever between January 2023 and December 2025. Blood cultures were processed using the BacT/ALERT system (bioMérieux, France), and isolates were identified by standard biochemical tests and VITEK 2 Compact (bioMérieux). Antimicrobial susceptibility testing (AST) was performed using the Kirby-Bauer disk diffusion method as per the Clinical and Laboratory Standards Institute (CLSI) guidelines. Bacterial whole-genome sequencing (WGS) was performed for two representative isolates, and resistance determinants were identified using NCBI databases.

Results

The median age of patients was 14.75 years with male predominance (73.3%). *Salmonella *Typhi accounted for 56.7% of isolates, followed by *Salmonella *Paratyphi A (43.3%). Fluoroquinolone resistance was observed in 63.3% of isolates. However, all isolates remained susceptible to ceftriaxone, azithromycin, chloramphenicol, cotrimoxazole and carbapenems. WGS revealed *gyrA* (S83F, D87N), *parC* (S80I) and *qnrS1*-mediated resistance in one isolate, while another demonstrated efflux-mediated resistance without quinolone resistance-determining region (QRDR) mutations.

Conclusion

The study highlights persistent fluoroquinolone resistance among *Salmonella* isolates causing enteric fever, alongside preserved susceptibility to cephalosporins, azithromycin and conventional first-line drugs. Continuous antimicrobial surveillance and genomic monitoring are essential to guide effective therapy and antimicrobial stewardship.

## Introduction

Enteric fever is a bacterial disease caused by *Salmonella enterica* serovar Typhi and *Salmonella enterica* serovar Paratyphi A, B and C [1]. Every year, approximately 9.3 million new cases of typhoid occur globally, causing 56,000 to 180,000 deaths [2]. It remains a major public health problem in low- and middle-income countries, especially in South and Southeast Asia, where inadequate sanitation, unsafe water supplies and overcrowding facilitate transmission [3]. The typical clinical presentation of the disease ranges from prolonged febrile illness with nonspecific symptoms to grave complications such as intestinal perforation, gastrointestinal haemorrhage, encephalopathy and septicaemia [4].

Historically, first-line antimicrobials for treating enteric fever included ampicillin, chloramphenicol and cotrimoxazole. However, the emergence of multidrug-resistant (MDR) typhoidal *Salmonella* strains, defined by combined resistance to the three agents mentioned, in the late 1980s and 1990s led to widespread therapeutic failure [5]. This probably triggered the extensive use of fluoroquinolones due to their excellent intracellular penetration and clinical efficacy [6]. The widespread and injudicious use of fluoroquinolones subsequently resulted in the emergence of quinolone resistance, most notably nalidixic acid-resistant *S. *Typhi (NARST), which is associated with reduced quinolone susceptibility and poor clinical response [7]. Consequently, *S. *Typhi and *S.* Paratyphi were listed as the World Health Organization (WHO) priority pathogens for antimicrobial resistance (AMR) surveillance [8].

The burden of fluoroquinolone resistance in enteric fever is particularly alarming in India. Data from the Indian Council of Medical Research Antimicrobial Resistance Surveillance Network (ICMR-AMRSN) 2024 report indicate that only approximately 1.3% of *Salmonella* isolates remain fully susceptible to fluoroquinolones, highlighting the limited role of this drug class in empirical therapy [9]. Currently, the first-line drugs recommended by the WHO for treatment of enteric fever include ciprofloxacin, ceftriaxone and azithromycin [10]. However, the recent emergence and spread of extensively drug-resistant (XDR) *Salmonella* Typhi strains, particularly from Asian countries, has further narrowed therapeutic options. XDR strains exhibit resistance to first-line agents, fluoroquinolones and third-generation cephalosporins, posing a serious threat to effective disease management and highlighting the urgent need for ongoing surveillance, antimicrobial stewardship and genomic characterisation of circulating strains [11].

Limited data integrating antimicrobial susceptibility and genomic resistance mechanisms among *Salmonella* isolates are available from Eastern India. The present study aimed to evaluate the clinical profile, antimicrobial susceptibility patterns, along with genomic characterisation of selected *Salmonella* isolates causing enteric fever at a tertiary care centre from Eastern India.

## Materials and methods

This was a retrospective, observational study conducted at a tertiary care centre in Eastern India from January 1, 2023, to December 31, 2025, including 30 patients with blood culture-confirmed enteric fever. The Institutional Ethics Committee approved the study.

Blood samples of patients with febrile illness received from various clinical departments were inoculated into BacT/ALERT blood culture bottles (bioMérieux, France). The bottles flagged as positive were further subjected to Gram staining, and subculturing was performed on blood agar and MacConkey agar. Isolates were identified based on colony morphology, Gram stain characteristics, standard biochemical tests, VITEK 2 Compact (bioMérieux, France) and confirmed by serotyping with *Salmonella* antisera. Antimicrobial susceptibility testing (AST) was carried out using the Kirby-Bauer disk diffusion method on Mueller-Hinton agar, in accordance with the Clinical and Laboratory Standards Institute (CLSI) guidelines. Bacterial whole-genome sequencing (WGS) was performed by Nucleome Informatics Private Limited (Hyderabad, Telangana, India) for two representative isolates, and resistance determinants were identified using NCBI databases.

Demographic and clinical details were retrieved retrospectively from hospital records. The clinical parameters analysed included age, sex, symptoms at presentation, documented co-infections (dengue, malaria, leptospirosis and scrub typhus), mode of management (outpatient or inpatient) and final clinical outcome (recovery or death). History of complications, such as gastrointestinal bleeding, intestinal perforation, hepatitis, septicaemia and neurological manifestations, were documented. The laboratory parameters that were analysed included total and differential leukocyte counts, liver enzyme activities such as aspartate aminotransferase (AST), alanine aminotransferase (ALT), and alkaline phosphatase (ALP), C-reactive protein (CRP) level, and serum urea and creatinine levels at admission. These parameters were evaluated to assess the haematological and biochemical profile of patients with blood culture-confirmed enteric fever. Data were entered into Microsoft Excel (Redmond, Washington, USA) and analysed using descriptive and non-parametric statistical methods. Continuous variables are expressed as median with interquartile range (IQR) or range, as appropriate, due to non-normal distribution and small sample size. Categorical variables are expressed as frequencies and percentages.

## Results

A total of 30 patients with blood culture-confirmed enteric fever were included in the study. The median age of patients was 14.75 years (range: 3 months-57 years), with a male predominance of 22 (73.3%). The majority (93.3%) of the patients were managed after hospitalisation, while only two patients were treated on an outpatient basis. The median duration of hospital admission was nine days (range: 2-45 days). The median duration of hospital admission did not differ significantly between patients infected with *S.* Typhi and *S.* Paratyphi (Mann-Whitney U test, U = 90.5, p = 0.814). Fever was present in all the patients at presentation. The mean duration of fever was 6.0 ± 1.9 days. Headache and myalgia were the next most frequent symptoms and were noted in 26 (86.7%) patients. Other symptoms, including abdominal pain (73.3%), anorexia (56.7%) and diarrhoea (23.3%), were also noted among the patients, and summarised in Table 1. Clinical improvement occurred in all patients.

**Table 1:** Symptoms of patients with enteric fever on presentation

Clinical features	Frequency
Fever	30 (100%)
Headache and myalgia	26 (86.7%)
Abdominal pain	23 (73.3%)
Anorexia	17 (56.7%)
Nausea/vomiting	11 (36.67%)
Diarrhoea	7 (23.3%)

*S.* Typhi was identified as the most frequent causative agent in 17 (56.7%) of the patients, followed by *S.* Paratyphi A in 13 (43.3%) cases. Leukocyte counts varied, with six (20%) patients demonstrating leukopenia and 23 (76.7%) showing normal counts. The median CRP level was 85 mg/L (range: 30.9-378.8 mg/L). The median serum AST, ALT, and ALP activities, and urea and creatinine levels were 95.5 (IQR: 60-149.25) U/L, 67.5 (IQR: 40.25-115.75) U/L, and 115.5 (IQR: 79.25-135) U/L, and 21 (IQR: 18-29) mg/dL and 0.74 (IQR: 0.60-0.89) mg/dL, respectively. The serological test for *S.* Typhi IgM antibodies was positive in two (6.7%) cases, whereas it was negative in 20 (66.7%) cases and data were not available for eight patients. Coinfection with scrub typhus, leptospirosis, dengue and malaria was not detected in any of the patients.

Antimicrobial susceptibility patterns

Among the 17 *S. *Typhi isolates, 11 (64.7%) were susceptible to ampicillin, and six (35.3%) isolates were susceptible to ciprofloxacin. Among the 13 *S.* Paratyphi A isolates, six (46.2%) were susceptible to ampicillin, and five (38.5%) were susceptible to ciprofloxacin. All 30 isolates were susceptible to amoxicillin-clavulanate, piperacillin-tazobactam, ceftriaxone, cefepime, azithromycin, chloramphenicol, cotrimoxazole, imipenem and meropenem. The resistance pattern of the included isolates is presented in Table 2.

**Table 2:** Antimicrobial susceptibility pattern of the enteric fever isolates S: susceptible; I: intermediate susceptible; R: resistant

Antimicrobial agent	*Salmonella *Typhi isolates (n=17)			*Salmonella *Paratyphi A (n=13)		
	S	I	R	S	I	R
Ampicillin	11 (64.7%)	-	6 (35.3%)	6 (46.2%)	-	7 (53.8%)
Amoxicillin-clavulanate	17 (100%)	-	-	13 (100%)	-	-
Piperacillin-tazobactam	17 (100%)	-	-	13 (100%)	-	-
Ceftriaxone	17 (100%)	-	-	13 (100%)	-	-
Cefepime	17 (100%)	-	-	13 (100%)	-	-
Ciprofloxacin	6 (35.3%)	3 (17.6%)	8 (47%)	5 (38.5%)	-	8 (61.5%)
Levofloxacin	8 (47%)	1 (5.9%)	8 (47%)	7 (53.8%)	-	6 (46.2%)
Azithromycin	17 (100%)	-	-	13 (100%)	-	-
Chloramphenicol	17 (100%)	-	-	13 (100%)	-	-
Cotrimoxazole	17 (100%)	-	-	13 (100%)	-	-
Imipenem	17 (100%)	-	-	13 (100%)	-	-
Meropenem	17 (100%)	-	-	13 (100%)	-	-

Whole-genome sequencing findings

WGS analysis of two representative *S.* Typhi isolates (X and Y) was performed to evaluate the mechanisms of fluoroquinolone resistance among the isolates. Isolate X harboured target site mutations in *gyrA* (S83F, D87N) and *parC* (S80I), along with the plasmid-mediated *qnrS1* gene, conferring high-level resistance through combined target alteration and efflux pump activation. In contrast, the isolate Y lacked *gyrA*/*parC* mutations and demonstrated efflux-mediated resistance only, as shown in Table 3.

**Table 3:** Genomic determinants of fluoroquinolone resistance in two representative Salmonella Typhi isolates

Resistance determinant	Isolate X	Isolate Y
*gyrA* mutations	S83F, D87N	Absent
*parC* mutations	S80I	Absent
Plasmid-mediated quinolone resistance (*PMQR*) genes	qnrS1 present	Not detected
Predominant resistance mechanism	Target alteration and efflux pumps	Efflux pumps

## Discussion

The present study describes the clinical profile, antimicrobial susceptibility patterns and genomic mechanisms of fluoroquinolone resistance among blood culture-confirmed enteric fever cases from a tertiary care centre. Despite the small sample size, the study provides valuable insights into evolving resistance trends in *Salmonella* spp. Enteric fever predominantly affects adolescents and young adults, with a median age of 14.75 years and male predominance, consistent with the findings by Shafqat et al [12]. The higher incidence among adolescents may relate to food habits and community exposure, while male predominance may reflect differences in healthcare-seeking behaviour or exposure risk.

Fever was universal at presentation, with generalised weakness and myalgia being the most common accompanying symptoms, highlighting the nonspecific presentation of enteric fever. Gastrointestinal symptoms, such as nausea, vomiting, abdominal pain, anorexia and diarrhoea, were variably present, necessitating microbiological confirmation due to overlap with other tropical febrile illnesses. These findings are consistent with those presented in the systematic review by Azmatullah et al [13]. No complications or atypical presentations were observed, and all patients recovered following timely diagnosis and appropriate antimicrobial therapy.

The inflammatory response in enteric fever triggers release of interleukin-6 and other cytokines, leading to increased hepatic synthesis of CRP. However, the wide variation in CRP levels suggests limited specificity, necessitating interpretation alongside clinical and microbiological findings. Etouke et al. reported a significant association between elevated CRP levels and enteric fever [14]. The low positivity rate of *S.* Typhi IgM serology in culture-confirmed cases highlights the limited sensitivity of serological tests and reinforces blood culture as the diagnostic gold standard, particularly during early disease. Sam et al. reported a sensitivity and a specificity of approximately 35% and 45%, respectively, of Typhidot, a rapid serological test [15]. 

*S. enterica* serovar Typhi accounted for the majority of enteric fever cases, followed by *S. enterica* serovar Paratyphi A. This distribution is consistent with the national trends given by ICMR-AMRSN 2024 [9]. Fluoroquinolone susceptibility was seen in 11 (36.7%) *Salmonella* isolates. A similar study from Kolkata reported fluoroquinolone resistance in 80% of *S.* Typhi and 90% of *S.* Paratyphi A isolates [16].

Fluoroquinolone resistance in *Salmonella* is mediated by mutations in quinolone resistance-determining regions (QRDRs) of *gyr* and *par* genes, along with plasmid-mediated mechanisms such as *qnr* genes and efflux pumps. WGS of two *S.* Typhi isolates revealed distinct resistance mechanisms. One isolate harboured *gyrA* (S83F, D87N) and *parC* (S80I) mutations along with *qnrS1* and efflux pumps, while the other showed only efflux-mediated resistance. Samajpati et al. reported decreased ciprofloxacin susceptibility associated with *gyrA* (S83F) mutations in *Salmonella* isolates from Kolkata [17].

Universal susceptibility to azithromycin, cephalosporins and re-emerging first-line agents, such as chloramphenicol and cotrimoxazole, is reassuring in this study. Similar studies by Varghese et al. and Chidambaram et al. also reported preserved susceptibility to ceftriaxone, azithromycin, chloramphenicol and cotrimoxazole among *Salmonella *isolates [18,19]. These findings suggest a possible reversal of resistance to older drugs due to reduced selective pressure following decreased use. Nevertheless, continuous antimicrobial surveillance remains essential.

The study was limited by its small sample size, retrospective single-centre design and whole-genome sequencing of only two representative isolates. The preserved susceptibility to conventional first-line agents may support reconsideration of these antibiotics in selected culture-confirmed cases.

## Conclusions

The study highlights persistent fluoroquinolone resistance among *Salmonella* isolates causing enteric fever, mediated by both chromosomal mutations and plasmid-mediated mechanisms. Preserved susceptibility to cephalosporins, azithromycin and conventional first-line drugs underscores the importance of continuous antimicrobial surveillance, culture and susceptibility guided therapy and antimicrobial stewardship.
